# COVID-19 rebound after oral treatment in a nursing home facility: A case series

**DOI:** 10.1016/j.imj.2022.10.003

**Published:** 2022-11-15

**Authors:** Alex P. Betrosian, Stavros M. Christou, Stavroula Kalathaki

**Affiliations:** a Evgenidion Hospital, Athens University, Papadiamantopoulou 20, 11527 Athens, Greece; b Kallisto Skilled Nursing Facility, Athens, Greece

**Keywords:** COVID-19, Infection, Paxlovid, Nursing facility

## Abstract

Paxlovid (nirmatrevir/ritonavir) is a 2 drug regimen taken together twice daily for 5 days was authorized for emergency use for nonhospitalized patients who are at risk for the progression of coronavirus disease (COVID-19). However, recurrence of symptoms 2–8 days after completing the treatment course has been recently recognized. In some cases patients tested negative on a direct SARS-CoV-2 viral test and then tested positive again (rebound COVID-19). The disease is mild and requires no additional antiviral treatment. Data are limited based on anecdotal case reports and few studies. According to the available data it is unclear if rebound symptoms are due to the drug treatment, drug resistance, re-infection or impaired immunity.

## 1 Introduction

Pfizer's Paxlovid is a 2-drug regimen (nirmatrelivir and ritonavir), taken together twice a day for 5 days. The Food and Drug Administration approved it for emergency use on December 22, 2021, in order to treating mild-to-moderate COVID-19 infection, in people who are in a higher risk for severe disease. Since its authorization, there was 90% effectiveness against severe outcome [1]. A rebound phenomenon has been recently reported and linked to the drug [2].

The CDC defines Paxlovid (COVID?) rebound, as when a patient who after receiving a full 5-day course of treatment, either has a reemergence of COVID symptoms or a positive test, 2 to 8 days after initial recovery, or a previous negative test. Some investigators have noticed a similar phenomenon with the drug molnupiravir, while others have found viral, and symptoms rebound in untreated COVID-19 infection [3,4].

The frequency of rebound COVID phenomenon remains unknown. Some studies have suggested that it occurs in <1% of patients receiving Paxlovid. However, most studies were conducted prior to the emergence of Omicron variant and employ a retrospective design. The Phizer clinical trial (EPIC-HF trial) reported that approximately 1%–2% of treated individuals had a rebound. Those rebounds were not statistically different from rebounds experienced in the placebo group [1]. In their retrospective analysis, Ranganath et al. showed that COVID rebound occurred in only 4 in 483 patients (0.8%) treated with nirmatrelivir/ritonavir [5].

The mechanism(s) of the Paxlovid rebound phenomenon remains unclear. Studies have suggested that neither drug resistance nor absence of neutralizing antibodies were likely causes of the recurrence of this undesirable condition [6]. It is tested whether the suppression by N/M viral replication can be resurged once the drug vanishes from the body, leading to high viral levels causing symptoms re-appearance. Whether longer courses of Paxlovid treatment would prevent most cases of rebound is also tested.

We report 2 cases of COVID-19 rebound, which had followed nirmatrelivir-ritonavir treatment in a nursing home facility.

## 2 Case study 1

An 85-year-old female with a past medical history of non-Hodgkin lymphoma, epilepsy, and recently performed colostomy due to colon cancer was admitted to a nursing facility for rehabilitation. She was vaccinated twice against COVID-19 with Phizer-BionNTech (22/03/21, 12/04/21) and boosted once (19/11/21). She was not on immune therapy. During her rehab course, she developed (had) low-grade fever, shortness of breath, sore throat, and hoarseness. An antigen test was positive for COVID-19 on 10/07/22 and she was placed on Paxlovid 500–100 twice daily on the same day (day 0). She required 2–3 L of oxygen with SaO_2_ 96%–97%. In the 5-day course was uncomplicated, she was apyretic and an antigen test which performed on 15/07/22 was negative. Two days later (17/7), she developed a low-grade fever and became positive for antigen test. Nasal SARS-CoV-2 reverse-transcriptase polymerase chain reaction (RT-PCR) was positive on 21/7 [RDRP (19), E GENE (18), N GENE (18)] with the cycle threshold (Ct) values of 33. The rebound phenomenon lasted until 23/07 and was mild and uncomplicated. The patient remains in a good condition following the rehabilitation program.

## 3 Case study 2

A 79-year-old man disabled by Parkinson disease (motor dysfunction, cognitive impairment, and psychosis) was admitted to a skilled nursing facility for long-term care. He was vaccinated twice against COVID- 19 with Phizer-BionNTech (14/04/21, 05/05/21) and boosted twice (24/11/21, 01/06/22). An antigen test was positive for COVID-19 on 15/07/22 after having symptoms of low-grade fever and dizziness. He was started on Paxlovid 500–100 mg therapy, twice daily for 5 days immediate after his diagnosis (day 0). His symptoms improved and the antigen test was negative 2 days after treatment. However, it became positive followed by mild symptoms 6 days after the end of treatment with positive both antigen and RT-PCR [RDRP (20), E GENE (18), N GENE (18)] with the cycle threshold (Ct) values of 28. He became negative with antigen and PCR tests 5 days later. The rebound course was mild and uncomplicated.

## 4 Discussion

COVID-19 disproportionately affects nursing home populations, due to the high proportion of frail older adults and those with underlying chronic conditions, resulting in hospitalization and high mortality rates [7]. In case of COVID-19 infection antiviral treatment is required, either in a hospital setting or within the nursing facility.

The 2 reported patients were assessed for a 5-day treatment with Paxlovid, which was started on day 0 of the diagnosis. Both patients had mild course, without any serious side-effects, and they became test-negative, 1–2 days after treatment, respectively. However, they developed rebound infection, confirmed by antigen and RT-PCR testing 2 and 6 days later vice versa. A CDC's May 24 health advisory noted that “a brief return of symptoms may be part of the natural history of SARS-CoV-2 infection in some persons, independent of treatment with Paxlovid and regardless of vaccination status.” Eventfully, this rebound phenomenon is limited because there are some anecdotal cases and few studies, which are published at this time [[2], [8], [9]]. According to the available data, it is unclear if rebound symptoms are due to the drug treatment, drug resistance, re-infection, or impaired immunity. In a recent study, Carlin et al., in their virologic and immunologic analysis of COVID-19 recrudescence, suggested that neither nirmatrelivir/ritonavir resistance nor absence of neutralizing antibody were a likely cause of recrudescence [6]. They concluded that the most likely possibility is insufficient drug exposure by individual pharmacokinetics, suggesting that either the drug is metabolized more quickly in some individuals or there is insufficient drug exposure, which has as a result longer treatment duration. However, according to CDC's advisory recent update, there is no evidence that additional treatment is required when a rebound is suspected.

This report is limited by the availability of only 2 comprehensively studied patients. There are several unanswered questions regarding incidence, clinical, and microbiologic course. Continuous surveillance, further research to determine the mechanism underlining COVID-19 rebounds and further studies that adjust treatment plans as necessary are required.
